# The Effects of a Single Transcranial Direct Current Stimulation Session on Impulsivity and Risk Among a Sample of Adult Recreational Cannabis Users

**DOI:** 10.3389/fnhum.2022.758285

**Published:** 2022-02-08

**Authors:** Herry Patel, Katherine Naish, Noam Soreni, Michael Amlung

**Affiliations:** ^1^Department of Psychiatry and Behavioural Neurosciences, McMaster University, Hamilton, ON, Canada; ^2^Peter Boris Centre for Addictions Research, St. Joseph’s Healthcare Hamilton & McMaster University, Hamilton, ON, Canada; ^3^Department of Applied Behavioral Science, The University of Kansas, Lawrence, KS, United States; ^4^Cofrin Logan Center for Addiction Research and Treatment, The University of Kansas, Lawrence, KS, United States

**Keywords:** cannabis, transcranial direct current stimulation, impulsivity, risk taking, delay discounting

## Abstract

Individuals with substance use disorders exhibit risk-taking behaviors, potentially leading to negative consequences and difficulty maintaining recovery. Non-invasive brain stimulation techniques such as transcranial direct current stimulation (tDCS) have yielded mixed effects on risk-taking among healthy controls. Given the importance of risk-taking behaviors among substance-using samples, this study aimed to examine the effects of tDCS on risk-taking among a sample of adults using cannabis. Using a double-blind design, 27 cannabis users [*M*(SD) age = 32.48 (1.99), 41% female] were randomized, receiving one session of active or sham tDCS over the bilateral dorsolateral prefrontal cortex (dlPFC). Stimulation parameters closely followed prior studies with anodal right dlPFC and cathodal left dlPFC stimulation. Risk-taking—assessed via a modified Cambridge Gambling Task—was measured before and during tDCS. Delay and probability discounting tasks were assessed before and after stimulation. No significant effects of stimulation on risk-taking behavior were found. However, participants chose the less risky option ∼86% of the trials before stimulation which potentially contributed to ceiling effects. These results contradict one prior study showing increased risk-taking among cannabis users following tDCS. There was a significant increase in delay discounting of a $1000 delayed reward during stimulation for the sham group only, but no significant effects for probability discounting. The current study adds to conflicting and inconclusive literature on tDCS and cognition among substance-using samples. In conclusion, results suggest the ineffectiveness of single session dlPFC tDCS using an established stimulation protocol on risk-taking, although ceiling effects at baseline may have also prevented behavior change following tDCS.

## Introduction

Cannabis use is relatively common among adults, with 22.2 million people in the United States reporting use in the past month on the 2015 National Survey on Drug Use and Health (Substance Use and Mental Health Administration, 2015), along with 3.18 million Canadians ages 15 and older reporting cannabis use within the past month in 2018 (Statistics Canada, 2020). Substance use has been linked to risk-taking behaviors (Hulka et al., 2013; Wang et al., 2013; Canavan et al., 2014; Yan et al., 2014). As such, examining risk-taking among cannabis users is critical given the significant prevalence of cannabis use among the general population. The results of some studies suggest a relationship between cannabis use and greater risk-taking tendencies on psychometrically validated measures of risk-taking such as the Iowa Gambling Task (Whitlow et al., 2004; Bolla et al., 2005) and the Balloon Analog Risk Task (Hanson et al., 2014). However, other studies have found minimal or no difference (Ramaekers et al., 2006; Metrik et al., 2012; Fischer et al., 2015). These discrepancies might be explained by heterogeneity of samples of “cannabis users” across different studies. Different studies use different inclusion and exclusion criteria related to cannabis use such as type of use (smoking, vaping, edibles, etc.), frequency (use per week, per typical session, etc.), and amount consumed (hits, joints, grams, etc.). Furthermore, not all studies use the same measures to assess use, thus leading to difficulties in directly comparing samples for risk-taking given the heterogeneity in cannabis users across studies.

The tendency to take risks is not confined to the context of cannabis use, with individuals who use other substances also displaying greater risk-taking tendencies than individuals who do not use substances on lab-based measures of risk-taking (e.g., Hulka et al., 2013; Wang et al., 2013; Canavan et al., 2014; Yan et al., 2014), such as the Cambridge Gambling Task (Rogers et al., 1999), Balloon Analog Response Task (Lejuez et al., 2002), and Probability Discounting Task (Koffarnus and Bickel, 2014). Risk-taking is operationalized within this context to be risky choices (i.e., choices that confer more uncertainty/increased loss in outcome) on behavioral tasks. Heightened risk-taking—whether it be in the realm of health, safety, or finance—can also be a barrier to recovery in individuals with substance use disorders (SUDs). As such, reducing risk-taking tendencies could enhance an individual’s capacity to reduce or abstain from substance use.

Neuromodulation techniques represent a promising method of altering risk-taking and other behaviors, but basic experimental research on the cognitive effects of these techniques is needed before these procedures can be developed into therapeutic interventions. One type of non-invasive brain stimulation that has been widely used in healthy and clinical populations is called transcranial direct current stimulation (tDCS). TDCS involves the delivery of a weak electrical current over the scalp, which causes temporary changes in neural excitability in the brain (Kuo et al., 2016). While the tDCS current is not strong enough to induce action potentials, it modulates the resting membrane potentials of neurons in the cortical area underlying the stimulating electrode, thereby making the neurons more or less likely to produce action potentials (Kuo et al., 2016). In this way, tDCS can be used to increase or decrease functioning in particular brain regions, which may also influence processing in larger brain networks. The direction of the neuronal changes induced by tDCS depends on the type of stimulation: anodal stimulation increases excitability by depolarizing the membrane potential, whereas cathodal stimulation decreases excitability through hyperpolarization of the membrane potential (Kessler et al., 2012).

Research in individuals who do not meet criteria for SUD found reduced risk-taking associated with anodal tDCS applied over the right dorsolateral prefrontal cortex (dlPFC), with the cathodal electrode positioned over the contralateral dlPFC (Fecteau et al., 2007; Cheng and Lee, 2015). More specifically, risk-taking was lower during anodal stimulation over the right dlPFC compared to during left anodal stimulation and sham stimulation. In SUDs, there is evidence of reductions in cravings for cannabis, alcohol, and tobacco following prefrontal tDCS (e.g., Boggio et al., 2008; Fregni et al., 2008; Den Uyl et al., 2015). However, the effects of this technique on risk-taking in people who use substances are unclear, with only one published study showing greater risk-taking behaviors among cannabis users who received anodal stimulation over the left or right dlPFC compared to those who received sham stimulation (Boggio et al., 2010). Other studies conducted among non-users show more inconclusive findings, where some find significant reductions in risk-taking (Pripfl et al., 2013; Gorini et al., 2014; Zheng et al., 2017; Nejati et al., 2018; Khaleghi et al., 2020) while others do not (Minati et al., 2012; Tremblay et al., 2014; Horvath et al., 2015; Lupi et al., 2017; Russo et al., 2017; Guo et al., 2018).

The primary objective of the current study was to examine effects of tDCS on risk-taking in a sample of individuals who use cannabis. Specifically, performance on a computerized Risk Task was assessed before and during anodal stimulation over the right dlPFC and cathodal stimulation over the left dlPFC. The secondary objective was to examine effects of tDCS stimulation on preferences on impulsive choice and on measures of subjective craving and affect.

## Methods

### Participants

Participants were adult cannabis users from the Hamilton, ON community. Inclusion criteria for cannabis users included: (1) between the ages of 18–55 years, (2) fluent English speakers, and (3) reporting using cannabis at least 3x/week. Exclusion criteria included: (1) contraindications for tDCS (e.g., history of epilepsy, seizures, medical devices/implants/metal in skull, history of brain trauma, pregnancy, etc.) and (2) diagnosis of bipolar disorder, episodes of mania/hypomania, and/or family history of bipolar disorder (first degree relative). Bipolar disorder or experience of mania/hypomanic episodes was excluded for due to potential induction of episodes via tDCS (Brunoni et al., 2017). Bipolar disorder symptoms were assessed with the Mini-International Neuropsychiatric Interview (MINI) and any questionable cases were evaluated by the study psychiatrist (NS). Participants were also required to abstain from using cannabis 12 h prior to their study appointment. Participants received a $40 gift card in compensation. This study was approved by the Hamilton Integrated Review Ethics Board (HiREB #4826), and all participants provided informed consent.

Twenty-nine cannabis users were enrolled in the study, from which two participants were excluded for failing to meet tDCS manipulation check (i.e., reporting that they did not believe they received stimulation in the active condition or correctly determining that they received sham stimulation). Of note, a group of non-users were also recruited, but the sample size was ultimately too small due to SARS-CoV-2 pandemic interruptions to permit data analyses^1^.

### Brain Stimulation Protocol

Direct current was delivered by a commercially available, battery-driven, constant current stimulator (***n***euroConn DC-Stimulator PLUS, neuroCare Group GmbH, Germany). Participants were randomly assigned to receive one session of either active (**n** = 15) or sham (**n** = 12) stimulation. Stimulation condition was double-blinded using “study mode” on the NeuroConn device; experimenters running the sessions were provided with a pre-set stimulation code in an individual envelope for each participant. The codes were prepared by the principal investigator (MA) who was not directly involved with stimulation sessions. Direct current was delivered via two rubber electrodes inserted in two saline soaked sponge pads (5 cm × 7 cm; 35 cm^2^) positioned bilaterally over dlPFC and held in place using rubber straps. Placement of electrodes was determined using the Beam F3 Locator Tool (Beam and Borckardt, 2010), which allows for the quick location of the F3 and F4 electrode positions on the 10–20 EEG system. Anodal stimulation was delivered over the right dlPFC (F3) and cathodal stimulation was delivered over the left dlPFC (F4) for all participants. Active and sham stimulation parameters were identical to those reported by Fecteau et al. (2007). In the active condition, a current of 2 mA was applied for 15 min, with the current increasing from 0 to 2 mA in the first 30 s of stimulation (ramp-up). In the sham condition, participants received the initial ramp-up from 0 to 2 mA current but the current was stopped after 30 s.

### Measures

Participants completed a variety of self-report questionnaires assessing demographics, verbal intelligence, impulsivity, cannabis use disorder symptoms, reasons for cannabis use, and use of nicotine (cigarettes smoked per day) and alcohol [Alcohol Use Disorders Identification Test (AUDIT); Saunders et al., 1993]. Refer to Supplementary Material for self-report measure descriptions. Participants completed behavioral tasks assessing delay and probability discounting, cannabis craving scales, and affect scales pre- and post-stimulation. Risk-taking, however, was assessed prior to and during stimulation.

#### Risk Task

The Risk Task involves gambling (little to no requirements of strategy and working memory) to assess decision-making (Rogers et al., 1999). The version used here was based on previous tDCS studies (Fecteau et al., 2007; Boggio et al., 2010); see Supplementary Figure 2. Participants underwent 100 trials (∼15 min to complete) in which six boxes were presented horizontally along the top of the screen (in varying ratios of pink or blue). On each trial, participants decided whether they believe a “winning token” is hidden behind a pink or a blue box. For correct guesses, they gained points; incorrect guesses lost points. The aim was to gain as many points as possible. However, two factors vary across the trials: (1) ratio of pink-to-blue boxes (5:1, 4:2, or 3:3 varied between trials) and (2) gain or loss amount (potential reward associated with each option in the ratios: 90:10, 80:20, 70:30, or 60:40). For example, on a 4:2 color ratio and 80:20 gain/loss amount trial, participants would be presented with an array of 4 blue and 2 pink squares associated with 20 and 80 points, respectively. If participants chose blue and the winning token was behind blue, they would earn 20 points but would lose 20 points if they were wrong. Alternatively, if they chose pink, they would earn 80 points if correct and lose 80 points if they were incorrect. The task was administered to participants twice, once before stimulation and once during stimulation to assess for stimulation-related changes.

#### Delayed Reward and Probability Discounting

Discounting of delayed and probabilistic rewards was assessed prior to and following stimulation, using two versions of the 5-trial adjusting delay discounting task and probability discounting task for $100 and $1000 rewards (Koffarnus and Bickel, 2014), the order of which was randomized across participants. On each of five trials for the delay discounting task, participants indicated whether they would rather receive a fixed larger amount of money ($100 or $1000) at a specific point in the future that varied across trials, or half the amount of money immediately ($50 or $500). The probability discounting task is essentially identical to the delay discounting one, except that the larger reward is probabilistic (i.e., 50% chance). Based on the choices across the five trials, the Effective Delay 50% (ED_50_) is estimated, and the inverse of this value is equivalent to the discount rate (e.g., *k* = 1/ED_50_) and probability discounting rate (e.g., *h* = 1/EP_50_) traditionally used in delay discounting research. *K*-values and *H*-values were log-transformed prior to analysis. As such, following log transformation, more positive *K*-values represent increased preferences for the immediate reward (i.e., greater impulsive choice), and more positive *H*-values represent increased preferences for the certain reward.

### Data Analysis

Variables were inspected for outliers (Zs > 3.29); no outlier values were identified. Repeated measures ANOVAs were conducted to assess changes in Risk Task performance, delay, and probability discounting across stimulation type. Outcome variables for Risk Task performance included percent safe choices: (1) across all trials, (2) on 4-to-2 box ratio trials, and (3) on 5-to-1 box ratio trials. Reaction times on the Risk Task were also examined using ANOVAs. Outcome variables for delay and probability discounting log transformed *k*-values (delay discounting) and *h*-values (probability discounting) for two versions of the task ($100 and $1000) administered pre- and post-stimulation. Finally, repeated measures ANOVAs were run to assess changes in affect and craving pre- and post-stimulation.

## Results

### Descriptive Statistics

The final sample consisted of 27 participants (40.7% female; 92.6% White). Independent samples *t*-tests revealed no significant differences in demographic and clinical variables across stimulation type, as such, no covariates were included in the repeated measures ANOVAs. Refer to Table 1 for sample characteristics.

**TABLE 1:** Sample characteristics stratified by stimulation type.

	Final sample (*n* = 27)	Active stim (*n* = 15)	Sham stim (*n* = 12)	*df*	*t/*χ *^2^*	*p*
Age	32.48 (1.99)	32.13 (11.19)	32.92 (11.23)	25	0.18	0.86
Sex	40.7%	46.7%	33.3%	1	0.49	0.48
Income	$37,500	$37,500	22,500	7	5.07	0.65
Education	14.30 (0.99)	14.33 (2.77)	14.25 (2.26)	25	–0.08	0.93
Verbal intelligence	30.85 (0.92)	30.93 (4.10)	30.75 (4.07)	25	–0.12	0.91
Cigarettes/day	5.71 (0.02)	7.15 (9.18)	4 (8.27)	22	–0.88	0.39
AUDIT total	5.93 (0.64)	5.2 (5.35)	6.83 (5.59)	25	0.77	0.44
AUDIT ≥ 8	18.5%	13.3%	25%	1	0.60	0.44
**Cannabis use characteristics**						
CUDIT total	12.81 (5.78)	12.93 (4.82)	12.67 (7.02)	25	–0.12	0.91
CUDIT ≥ 8	81%	86.7%	75%	1	0.60	0.44
CUDIT ≥ 13	59.3%	40.0%	33.3%	1	0.00	0.93
Age at first use	15.92 (3.33)	16.93 (3.93)	14.75 (2.05)	24	1.73	0.10
Age at regular use	20.04 (4.29)	20.93 (5.24)	19.00 (2.70)	24	1.15	0.26
Typical day use	1.62 (1.07)	1.65 (0.89)	1.57 (1.28)	24	0.19	0.84
**Psychiatric symptom characteristics**						
Anxiety symptoms	5.44 (0.70)	5.27 (4.18)	5.67 (4.79)	25	0.23	0.82
Depressive symptoms	5.26 (0.81)	4.27 (3.56)	6.5 (5.13)	25	1.34	0.19
**Impulsivity characteristics**						
Negative urgency	2.23 (0.14)	2.13 (0.76)	2.35 (0.65)	25	0.80	0.43
Lack of Premeditation	1.81 (0.12)	1.72 (0.44)	1.92 (0.59)	25	1.01	0.32
Lack of Perseverance	1.87 (0.13)	1.93 (0.43)	1.79 (0.66)	25	–0.67	0.51
Sensation seeking	2.70 (0.19)	2.65 (0.62)	2.77 (0.73)	25	0.46	0.65
Positive urgency	1.75 (0.16)	1.6 (0.57)	1.94 (0.58)	25	1.53	0.14

*All values presented are means (standard deviations) except for sex (percentage of females) and income (median income). Chi-square statistics are presented for sex and income only. Sex, sex at birth; Education, years of education; Verbal Intelligence, sum score on Shipley-Verbal; AUDIT, Alcohol Use Disorders Identification Test; CUDIT, Cannabis Use Disorder Identification Test, scores of ≥8 indicate hazardous use and scores of ≥13 indicate potential cannabis use disorder; Anxiety Symptoms, sum score of Generalized Anxiety Disorders Scale; Depressive Symptoms, sum score on depression module of Patient Health Questionnaire; Negative Urgency, Lack of Premeditation, Lack of Perseverance, Sensation Seeking, Positive Urgency, subscales of the Short UPPS-P scale.*

### Effects of Transcranial Direct Current Stimulation on Risk-Taking

There were no significant main effects of time or stimulation type nor any significant interactions for all outcome variables (η_*p*_^2^ = 0.001 to 0.1). Refer to Table 2 for full statistical results and Supplementary Table 1 for means (standard error) for active/sham groups at each time point. Supplementary Figure 4 presents individual-subject data and means for each group at each time point. Although the sham group, on average, appeared to make fewer safe choices at baseline, this difference was not statistically significant (*p* = 0.08). Also of note, most active group participants made >80% safe choices at baseline which may have constrained sensitivity to detect significant improvement during tDCS. In examining the effects of tDCS on reaction time within the risk-taking task, we found a significant main effect of time (η_*p*_^2^ = 0.49 to 0.67) but not stimulation type or any significant interactions (see Supplementary Table 2).

**TABLE 2:** Repeated measures ANOVA results for risk-taking.

	Overall % safe choices^a^			4:2 % safe choices^a^			5:1 % safe choices^a^		
			
Source	*F*	*p*	η *_*p*_^2^*	*F*	*p*	η *_*p*_^2^*	*F*	*p*	η *_*p*_^2^*
Time (T)	1.29	0.27	0.049	2.70	0.113	0.098	0.08	0.78	0.003
Stimulation Type (ST)	2.52	0.13	0.091	2.83	0.105	0.102	2.04	0.17	0.075
T × ST	0.25	0.62	0.010	0.46	0.504	0.018	0.03	0.86	0.001

*^a^df = 1, 25.*

### Effects of Transcranial Direct Current Stimulation on Delay and Probability Discounting

There was a significant main effect of time on the delay discounting rate [*F*(1,22) = 13.55, *p* = 0.001, η_*p*_^2^ = 0.381] for the $1000 version. Furthermore, there was a significant time × stimulation type interaction [*F*(1,22) = 6.80, *p* = 0.016, η_*p*_^2^ = 0.236] where delay discounting rate among individuals in the sham condition increased from pre-stimulation (*M* = −2.04, SE = 0.27) to post-stimulation (*M* = −1.53, SE = 0.30). However, there were no significant main effects of time or stimulation type nor any significant interactions for all other outcome variables. Refer to Table 3 for full statistical results and Supplementary Table 1 for means (standard error) for active/sham groups at each time point.

**TABLE 3:** Repeated measures ANOVA results for delay and probability discounting.

	Delay discounting						Probability discounting					
		
	100 *k*^a^			1000 *k*^b^			100 *h*^c^			1000 *h*^c^		
				
Source	*F*	*p*	η *_*p*_^2^*	*F*	*p*	η *_*p*_^2^*	*F*	*p*	η *_*p*_^2^*	*F*	*p*	η *_*p*_^2^*
Time (T)	2.59	0.12	0.097	13.55	0.001	0.381	0.01	0.95	0.000	0.11	0.74	0.005
Stimulation Type (ST)	0.04	0.84	0.002	0.69	0.42	0.030	0.08	0.79	0.003	0.10	0.75	0.004
T × ST	0.55	0.47	0.022	6.80	0.016	0.236	1.59	0.22	0.065	0.00	1.00	0.000

*^a^df = 1, 24; ^b^df = 1, 22; ^c^df = 1, 23.*

### Effects of Transcranial Direct Current Stimulation on Affect and Cannabis Craving

Scales of affect included: tense-calm, sad-happy, nervous-relaxed, bored-excited, stressed-serene, and depressed-elated. There were no significant main effects of time or stimulation type nor any significant interactions for any affect variables. Refer to Supplementary Table 3 for full statistical results.

Scales assessing cannabis craving included four dimensions: use, craving, urge, and desire. There were no significant main effects of group or stimulation type nor any significant interactions for any craving variables. Refer to Supplementary Table 4 for full statistical results.

## Discussion

The purpose of this study was to examine effects of a single session of tDCS on risk-taking before and during anodal stimulation over the right dlPFC and cathodal stimulation over the left dlPFC in a sample of individuals who reported using cannabis regularly. A secondary objective was to examine the effects of tDCS over the right dlPFC on delay discounting, probability discounting, subjective cannabis craving, and affect. For the primary objective, we found no significant effects of tDCS on any dependent variable on the Risk Task except for reaction time, where a significant main effect of time was found. However, this result, in combination with the lack of significant differences in percent safe choice can most likely be attributed to practice effect; participants were faster in responding the second time because they had already completed the task once before. For the secondary objectives, the only significant effects were a significant main effect of Time and Time × Stimulation Type interaction for the $1000 version of the delay discounting task. This effect was driven by significant increases in discounting rate following sham stimulation but not active stimulation. Generally speaking, this is the opposite pattern one would expect if tDCS was reducing impulsive behavior with anodal stimulation over the dlPFC. Given that no other studies have examined the effects of tDCS on delay discounting among either substance using or non-using samples, there is limited basis to compare these results to prior literature.

The present finding that tDCS did not significantly affect risk-taking behavior is generally consistent with numerous previous studies reporting similar non-significant effects (Minati et al., 2012; Fecteau et al., 2014; Horvath et al., 2015; Ye et al., 2015; Russo et al., 2017; Guo et al., 2018). A recent meta-analysis found a non-significant effect for unilateral (right or left) active tDCS over the dlPFC for risk-taking among healthy adults as compared to sham stimulation (Khaleghi et al., 2020). Of the ten studies included for unilateral stimulation, three studies reported an increase in risky behaviors and two studies reported no significant differences (Khaleghi et al., 2020). Among studies that did find significant differences in risk-taking, some studies did not compare performance within subjects (Nejati et al., 2018), while others used repeated stimulation (Gorini et al., 2014) or high-density arrays of multiple electrodes (Pripfl et al., 2013). Additionally, a prior systematic review examining the effects of tDCS on other cognitive domains found inconsistencies in methodology and results for tDCS (Tremblay et al., 2014), indicating that stimulation parameters may be key to see significant effects of tDCS on cognition.

To our knowledge, only one study to date examined the effects of tDCS on risk taking among cannabis users (Boggio et al., 2010). That study, with an identical single-session tDCS stimulation protocol, unexpectedly found increased risk taking among chronic cannabis users during active stimulation of left or right dlPFC compared to cannabis users who received sham stimulation. In contrast, the current study found no significant effects of active or sham tDCS on risk-taking among cannabis users. One of the potential reasons for this inconsistency could be the differences in samples where Boggio et al. (2010) reported approximately 5.5 uses/week among their sample, however, we assessed use in grams for a typical day of use. Furthermore, we administered the CUDIT-R whereas Boggio and colleagues did not, which speaks to the heterogeneity in cannabis user samples within the literature making direct comparisons across studies more difficult. Comparison to other findings in participants who use other substances is complicated by differences in tasks, stimulation protocol, and potential variation based on substance type. For example, among a sample of people who were recently abstinent from cocaine use who completed three tDCS sessions (left anodal dlPFC, right anodal dlPFC, and sham), left and right stimulation of the dlPFC was associated with decreases in risk-taking behavior as assessed by the Balloon Analogue Risk Task (BART) compared to the sham condition (Gorini et al., 2014). Lastly, Pripfl et al. (2013) examined the effects of high-density tDCS of the dlPFC on risk-taking among a sample of tobacco smokers. Left anodal stimulation of the dlPFC was associated with decreased risk taking among smokers in a “cold” (deliberative) version of the Columbia Card Task which is inconsistent with our results. Together with the findings from the current study, substantial heterogeneity in participant characteristics, risk-taking tasks, hemisphere of anodal vs. cathodal stimulation considerably obscures conclusions about the effects of dlPFC tDCS on risk-taking in substance use samples.

Given that we did not find any significant effects of tDCS on risk-taking, it is important to discuss potential alternative explanations for a null result beyond simply concluding that tDCS is ineffective. One such explanation is related to optimal performance on the Risk Task at baseline. Examining the distributions in Supplementary Figure 2 indicates the presence of ceiling effects for the Risk Task at baseline, particularly among the active stimulation group. With the exception of a single participant, baseline percentage of safe choices ranged from 84 to 100% in the active group (mean = 91%), increasing to 90–100% during stimulation (mean = 92%). Therefore, it is possible that participants in the active group were performing optimally at baseline and did not exhibit meaningful risk-taking behavior that could be influenced by tDCS. Previous studies using the same Risk Task as ours reported the percentage of safe choices among thirty-six healthy controls to range between 79 and 92% (Fecteau et al., 2007) and 87% of trials for the only study of twenty-five cannabis users (Boggio et al., 2010). Given our comparable percentage of safe choices across multiple studies with healthy controls and cannabis users, a lack of significant results could be due to the lack of sensitivity of the Risk Task measure in assessing the effects of tDCS on risk-taking behavior. Another consideration for null results is the small sample size which could have constrained power to detect significant effects. Although the groups were well matched on participant characteristics, small sample sizes may have resulted in suboptimal randomization of baseline risk-taking behavior between the groups.

Another factor that may have contributed to the present null findings is that participants only underwent a single session of tDCS stimulation. However, single-shot tDCS experiments are common (Boggio et al., 2010; Minati et al., 2012; Ye et al., 2015; Guo et al., 2018; Nejati et al., 2018), including the study by Boggio et al. (2010) that reported increased risk taking in cannabis users following a single episode of DLPFC stimulation. While it is plausible to speculate that reductions in risk taking among cannabis users may only occur following multiple stimulations (suggesting a potential dose-response effect), prior research largely does not support this hypothesis. First, Fecteau et al. (2014) examined the effects of five consecutive days of tDCS stimulation over right DLPFC in a sample of smokers. They observed a significant decrease in cigarettes smoked and increased rejection of cigarettes in a decision-making task, but performance on the Risk Task was not affected. Second, in a prior review of studies examining tDCS among samples with substance use disorders, multiple studies with repeated sessions of tDCS (∼2–5 sessions) found significant reductions in craving but no significant differences in risk-taking or decision making processes (Trojak et al., 2017). With the caveat that differences may exist between cannabis use and other substances, repeated exposure to tDCS may have beneficial effects on substance use behaviors (e.g., craving and consumption) but does not appear to have an additive effect on risk taking behavior.

The current study is not without its limitations and our results should be interpreted with caution. The most important limitation is the small sample size which could have contributed to low statistical power to detect significant effects. Regardless of small sample size, it is important to note the relatively small effect sizes (η_*p*_^2^ = 0.001–0.1) for performance on the Risk Task suggesting minimal (if any) effects of tDCS were present. In addition, while the 5-choice delay and probability discounting tasks have been shown to be sensitive to experimental manipulations in published studies (Bickel et al., 2016; Stein et al., 2017, 2018; Mellis et al., 2018), there is no published test-retest reliability data available for these tasks. Furthermore, individuals were asked to not use cannabis for at least 12 h prior to their appointment. Adherence to this was based on self-report and no biochemical measure was used to confirm. Thus, it is possible that individuals were experiencing residual effects which could have unintentionally impacted performance on the study measures. Another limitation is a lack of diversity in the sample, thus making it critical to examine whether significant effects of tDCS can be seen in underrepresented minority populations who also show disproportionately higher rates of cannabis use (Keyes et al., 2017). Finally, we were not able to include the comparison sample of non-users due to negative impact of the SARS-CoV-2 pandemic. A larger study that includes both cannabis and control groups using the same protocol is necessary to further resolve discrepancies across our study and that of Boggio et al. (2010).

In conclusion, we found no significant effects of tDCS over the dlPFC on risk-taking behavior among a sample of adult cannabis users, although we cannot completely rule out the possibility of ceiling effects in the active stimulation group. Our study adds to the growing inconclusive literature on the effects of tDCS on cognition among both healthy controls and substance using samples, particularly for single session tDCS (see quantitative review: Horvath et al., 2015). While tDCS has been shown to be effective for certain psychiatric disorders (i.e., major depressive disorder), it is premature to conclude any beneficial effects of single session tDCS on more complex cognitive processes such as risk-taking, executive functioning, among others.

## Data Availability Statement

The raw data supporting the conclusions of this article will be made available by the authors upon request, without undue reservation.

## Ethics Statement

The studies involving human participants were reviewed and approved by the Hamilton Integrated Review Ethics Board (HiREB Project #4826). The participants provided their written informed consent to participate in this study.

## Author Contributions

HP, KN, and MA were responsible for the design of the study. HP collected the data with oversight from NS and MA, and wrote the first draft of the manuscript. HP and MA were responsible for data analysis. KN, NS, and MA provided edits. All authors approved the final version.

## Conflict of Interest

The authors declare that the research was conducted in the absence of any commercial or financial relationships that could be construed as a potential conflict of interest.

## Publisher’s Note

All claims expressed in this article are solely those of the authors and do not necessarily represent those of their affiliated organizations, or those of the publisher, the editors and the reviewers. Any product that may be evaluated in this article, or claim that may be made by its manufacturer, is not guaranteed or endorsed by the publisher.

## Footnotes

1 Data collection for this study was prematurely terminated due to government shutdown of human participant research due to the SARS-CoV-2 pandemic. Given the close physical proximity of tDCS procedures, we were unable to resume data collection in a manner that was considered compliant with physical distancing requirements or placed unnecessary burden on participants.
