# Supplementary material for: The Effects of a Single Transcranial Direct Current Stimulation Session on Impulsivity and Risk Among a Sample of Adult Recreational Cannabis Users
Source: Front Hum Neurosci. 2022 Feb 8;16:758285. doi: 10.3389/fnhum.2022.758285 (PMC8861082; doi:10.3389/fnhum.2022.758285)
Supplement: Supplementary file 1 [file Data_Sheet_1.docx]

Supplementary Material

# Supplementary Information

## Self-Report Measure Descriptions

### Demographics

Demographic variables included age, sex, annual income, education and were assessed via self-report.

### Verbal Intelligence

Verbal intelligence was assessed via the verbal reasoning subscale of Shipley Institute of Living Scale. Participants were presented with forty words ranging in difficulty from easy to difficult. For each word, participants were presented with 4 corresponding words and asked to choose the synonymous corresponding words. The correct responses are summed as a representation of verbal intelligence score.

### Nicotine Use

Nicotine use was assessed via two-items from the Fagerstrom Test for Nicotine Dependence (FTND). One item assessed frequency of cigarette and e-cigarette use in the past 3 months based on a scale ranging from None to Multiple Times Daily. The other item was a free response in which participants were asked how many cigarettes they smoke per day. If participants did not smoke every day, then were asked to provide an average by dividing the number of cigarettes smoked in past month by 30 days.

### Alcohol Use Disorder Symptom Severity

Alcohol use frequency and alcohol-related problems were measured using the Alcohol Use Disorders Identification Test (AUDIT)^24^, a self-report screen assessing the frequency and severity of alcohol use. Scores on the AUDIT range from 0–40 and a cutoff of 8 or greater indicates clinically hazardous use.

### Cannabis Use Disorder Symptom Severity

The Cannabis Use Disorder Identification Test-Revised (CUDIT-R)^25^ assessed cannabis use frequency and related problems over the past 6 months. Scores on the CUDIT-R ranged from 0–32 and a cutoff of 8 or greater indicates hazardous use while a cutoff of 13 or greater indicates potential cannabis use disorder.

### Affect Scales

Visual analog scales ranging from -50 to 50 were used to assess affect for multiple ranges of emotion: (1) tense to calm, (2) sad to happy, (3) nervous to relaxed, (4) bored to excited, (5) stressed to serene, and (6) depressed to elated. The negative emotion was always assigned to -50 and the positive emotion was assigned to 50. Participants were instructed to place an X along the scale as they felt currently. Affect scales were administered twice: prior to stimulation and post-stimulation.

### Cannabis Craving Scales

Visual analog scales ranging from 0-100 (minimum to maximum) were to assess four aspects of craving in the current moment: (1) use, (2) craving, (3) urge, and (4) desire. Participants were instructed to place an X along the scale as they felt currently. Affect scales were administered twice: prior to stimulation and post-stimulation.

# Supplementary Figures and Tables

## Supplementary Figures

**
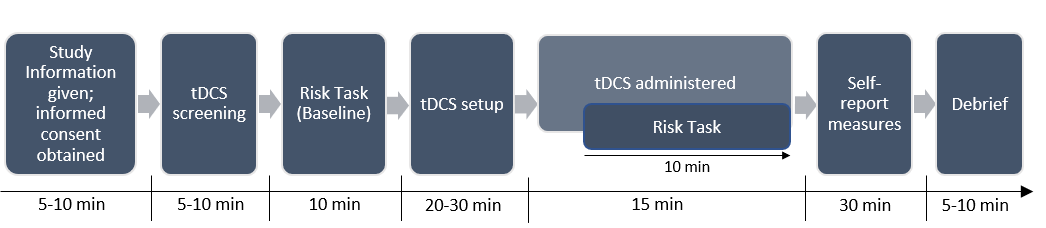
**

**Supplementary Figure 1.** Study Session Timeline.


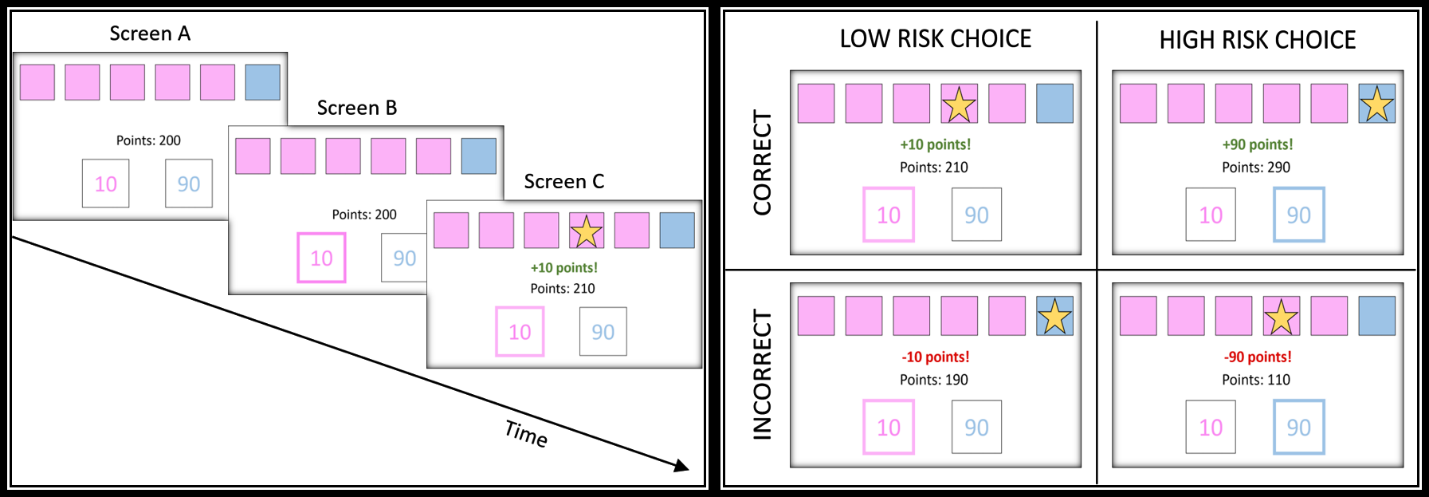


**Supplementary Figure 2. Risk Task Schematic.** *Left Panel* shows trial structure of a typical trial in the Risk Task. Screen A displays the ratio of pink to blue boxes (5:1 on the trial shown) and is displayed at the beginning of the trial. The number of points currently held by the participant is shown in the middle of the screen, and the points associated with the choices on the current trial are shown in the boxes at the bottom of the screen. Screen B is the screen displayed when the participant has selected an option; the highlighted box shows that, in this case, ‘pink’ was selected. Screen C shows the trial outcome; the winning token (the star) was hidden behind a pink box, so the participant gains 10 points. *Right Panel* shows the possible choices and outcomes for the trial shown in the *left panel.*


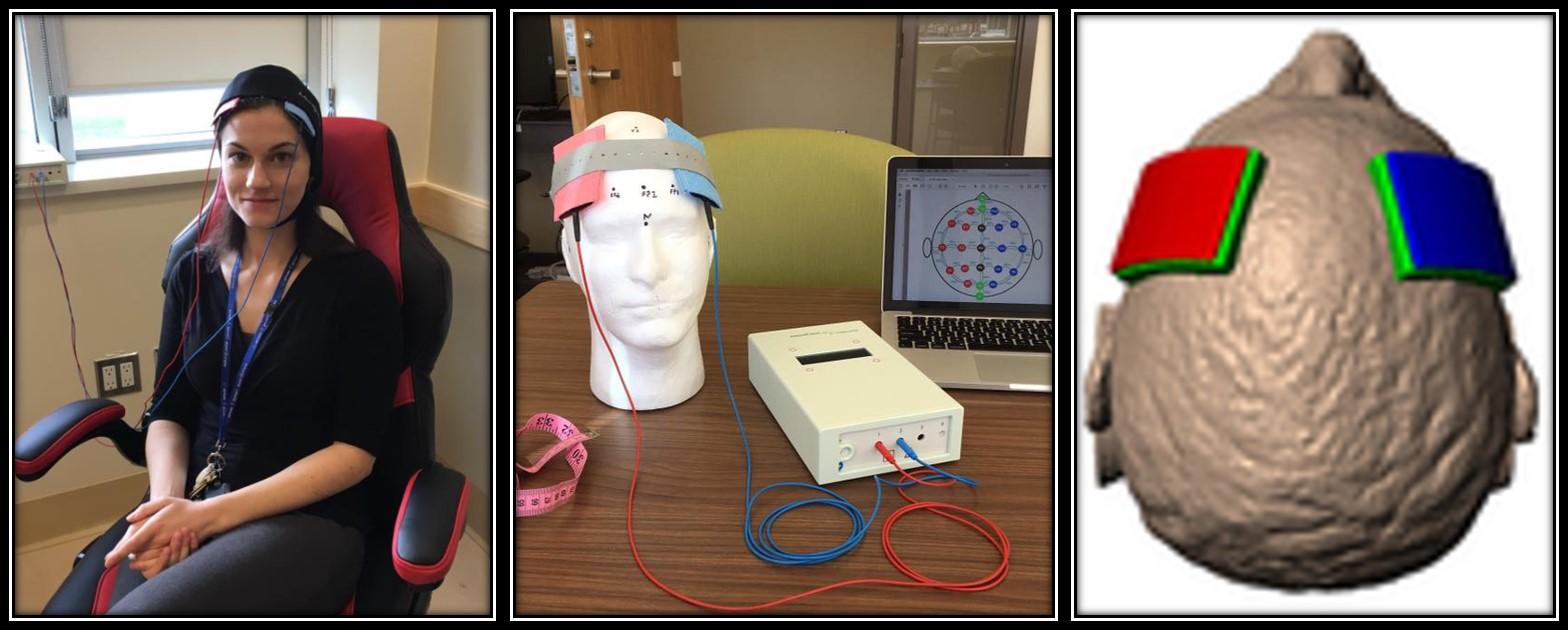

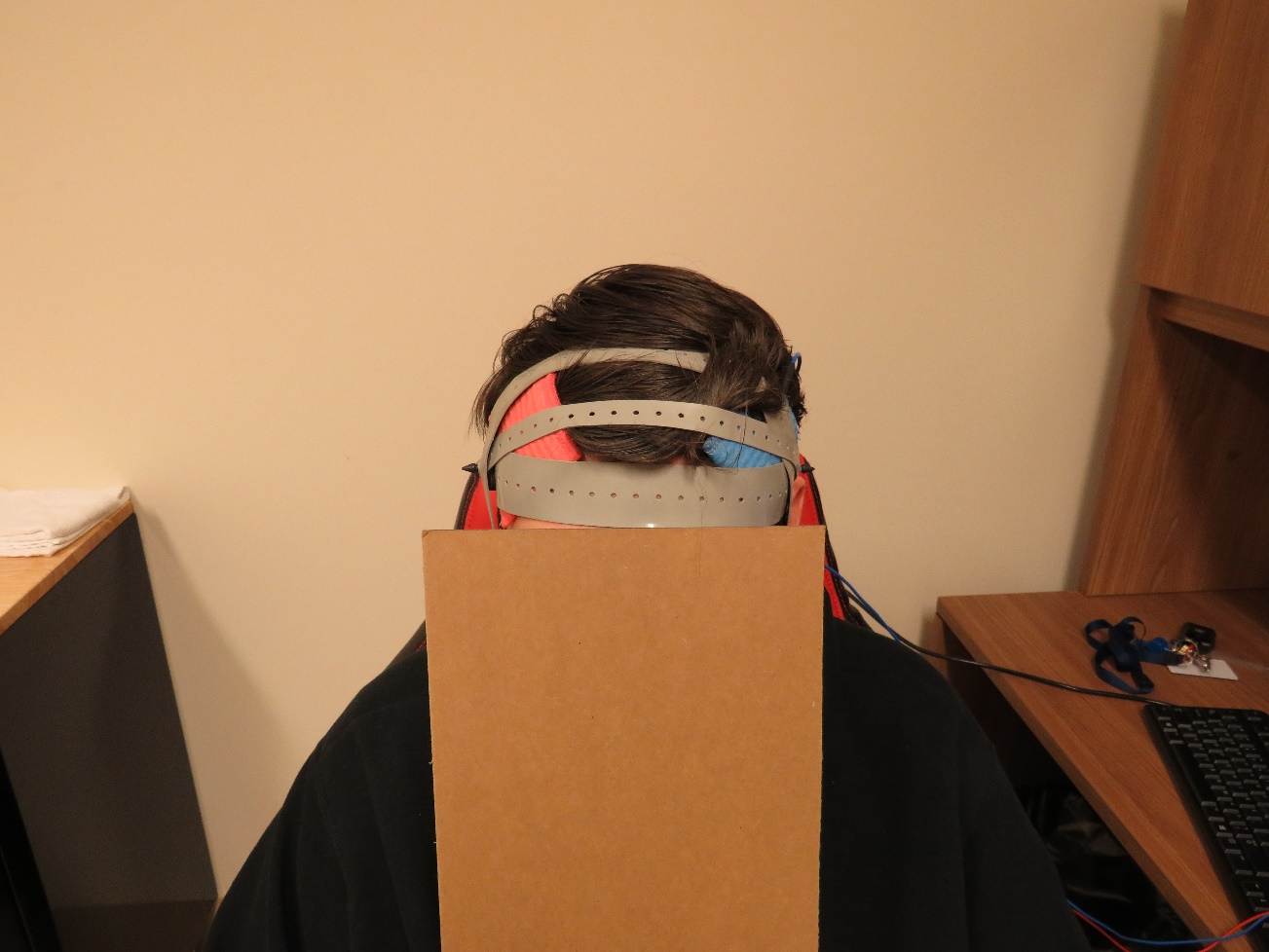


**Supplementary Figure 3. tDCS Electrode Placement Image.** Photo of the tDCS device (NeuroConn DC-Stimulator PLUS) set up in the Peter Boris Centre for Addictions Research depicting electrode placement over F3 and F4 on a dummy figurine, and an image of a participant (face obscured) depicting electrode positioning for bilateral stimulation (right [red] = anodal; left [blue] = cathodal) over the DLPFC.

**Supplementary Figure 4. Risk Task Performance by Stimulation Group and Time Point.**

Datapoints reflect individual participants in active and sham groups for baseline (pre) and during stimulation (stim). Horizontal line reflects group mean with standard error. Note, although several participants performed disproportionately worse than other participants in their respective groups, these data points did not meet statistical definition of outliers (Zs < 3.29) and were not excluded/winsorized prior to analysis.

## Supplemental Tables

**Supplemental Table 1. Descriptive Statistics for Each Dependent Measure of Interest Pre and Post/During Stimulation.**

|  | **Pre-Stimulation** | | **During/Post-Stimulation** | |
| --- | --- | --- | --- | --- |
|  | **Active** | **Sham** | **Active** | **Sham** |
| **Risk Taking** |  |  |  |  |
| **Overall % Safe Choices** | 0.91 (0.04) | 0.81 (0.04) | 0.92 (0.04) | 0.84 (0.06) |
| **4:2 % Safe Choices** | 0.89 (0.04) | 0.77 (0.04) | 0.91 (0.04) | 0.82 (0.06) |
| **5:1 % Safe Choices** | 0.93 (0.03) | 0.85 (0.04) | 0.94 (0.04) | 0.86 (0.05) |
| **Risk Taking Reaction Time (ms)** |  |  |  |  |
| **Overall** | 1713.46 (638.22) | 1346.36 (483.14) | 1199.84 (523.62) | 1060.96 (581.20) |
| **4:2** | 1772.61 (672.90) | 1338.17 (411.10) | 1228.81 (550.64) | 1095.48 (582.30) |
| **5:1** | 1657.36 (619.41) | 1355.02 (591.10) | 1171.88 (501.64) | 1027.22 (581.85) |
| **Delay Discounting** |  |  |  |  |
| **100k** | -1.57 (0.32) | -1.71 (0.22) | -1.49 (0.30) | -1.50 (0.27) |
| **1000k** | -2.06 (0.27) | -2.10 (0.22) | -2.05 (0.31) | -1.53 (0.24) |
| **Probability Discounting** |  |  |  |  |
| **100h** | 0.16 (0.18) | 0.15 (0.13) | 0.09 (0.13) | 0.21 (0.12) |
| **1000h** | 0.25 (0.11) | 0.20 (0.14) | 0.24 (0.10) | 0.19 (0.13) |
| **Affect Scales** |  |  |  |  |
| **Tense-Calm** | 31.27 (4.80) | 33.92 (4.18) | 28.13 (4.29) | 27.92 (7.40) |
| **Sad-Happy** | 27.53 (5.18) | 18.75 (7.20) | 25.53 (4.85) | 18.33 (6.42) |
| **Nervous-Relaxed** | 28 (4.72) | 31 (5.01) | 25.93 (4.20) | 23.42 (7.49) |
| **Bored-Excited** | 9.33 (4.57) | 12.58 (5.84) | 8.33 (4.62) | 6.33 (6.08) |
| **Stressed-Serene** | 20 (6.44) | 10.58 (6.99) | 20.47 (5.52) | 16.83 (7.33) |
| **Depressed-Elated** | 20.07 (4.61) | 7.58 (7.48) | 20.4 (3.58) | 11.25 (7.15) |
| **Craving Scales** |  |  |  |  |
| **Use** | 44.73 (8.06) | 44.17 (9.52) | 46.13 (7.11) | 37.42 (10.05) |
| **Craving** | 25.67 (6.58) | 34.5 (9.71) | 28.07 (6.57) | 31.75 (10.19) |
| **Urge** | 26.8 (6.23) | 36 (10.52) | 26.2 (6.09) | 31.5 (10.12) |
| **Desire** | 31.6 (6.15) | 39.5 (9.91) | 30.13 (6.26) | 36.42 (10.32) |
| Note: all values presented are means (standard error). | | | | |

**Supplemental Table 2. Repeated Measures ANOVA Results for Risk-Task Reaction Times for Overall Task, 4:2 Choice Block, and 5:1 Choice Block**

|  | **Overall % Safe Choices^a^** | | | **4:2 % Safe Choices^a^** | | | **5:1 % Safe Choices^a^** | | |
| --- | --- | --- | --- | --- | --- | --- | --- | --- | --- |
| **Source** | ***F*** | ***p*** | ***η_p_^2^*** | ***F*** | ***p*** | ***η_p_^2^*** | ***F*** | ***p*** | ***η_p_^2^*** |
| **Time (T)** | 38.363 | .000 | .605 | 24.065 | .000 | .490 | 52.244 | .000 | .676 |
| **Stimulation Type (ST)** | 1.478 | .235 | .056 | 1.915 | .179 | .071 | 1.081 | .308 | .041 |
| **T X ST** | 3.130 | .089 | .111 | 3.527 | .072 | .124 | 1.964 | .173 | .073 |
| Note: ^a^*df* = 1, 25. | | | | | | | | | |

**Supplemental Table 3. Repeated Measures ANOVA Results for Affect Scales**

|  | **Tense-Calm** | | | **Sad-Happy** | | | **Nervous-Relaxed** | | | **Bored-Excited** | | | **Stressed-Serene** | | | **Depressed-Elated** | | |
| --- | --- | --- | --- | --- | --- | --- | --- | --- | --- | --- | --- | --- | --- | --- | --- | --- | --- | --- |
| **Source** | ***F*** | ***p*** | ***η_p_^2^*** | ***F*** | ***p*** | ***η_p_^2^*** | ***F*** | ***p*** | ***η_p_^2^*** | ***F*** | ***p*** | ***η_p_^2^*** | ***F*** | ***p*** | ***η_p_^2^*** | ***F*** | ***p*** | ***η_p_^2^*** |
| **Time (T)** | 1.93 | .18 | .072 | 0.49 | .49 | .019 | 2.21 | .15 | .081 | 2.62 | .12 | .095 | 2.18 | .15 | .080 | 0.78 | .39 | .030 |
| **Stimulation Type (ST)** | 0.03 | .86 | .001 | 0.97 | .33 | .038 | 0.00 | .97 | .000 | 0.01 | .93 | .000 | 0.53 | .48 | .021 | 1.99 | .17 | .074 |
| **T X ST** | 0.19 | .67 | .008 | 0.21 | .65 | .008 | 0.72 | .40 | .028 | 1.37 | .25 | .052 | 1.61 | .22 | .061 | 0.54 | .47 | .021 |
| Note: *df* = 1, 25. | | | | | | | | | | | | | | | | | | |

**Supplemental Table 4. Repeated Measures ANOVA Results for Craving Scales.**

|  | **Use** | | | **Craving** | | | **Urge** | | | **Desire** | | |
| --- | --- | --- | --- | --- | --- | --- | --- | --- | --- | --- | --- | --- |
| **Source** | ***F*** | ***p*** | ***η_p_^2^*** | ***F*** | ***p*** | ***η_p_^2^*** | ***F*** | ***p*** | ***η_p_^2^*** | ***F*** | ***p*** | ***η_p_^2^*** |
| **Time (T)** | 1.20 | .28 | .046 | 0.01 | .91 | .001 | 2.63 | .12 | .095 | 1.33 | .26 | .050 |
| **Stimulation Type (ST)** | 0.15 | .70 | .006 | 0.30 | .59 | .012 | 0.41 | .53 | .016 | 0.40 | .53 | .016 |
| **T x ST** | 2.79 | .11 | .100 | 2.89 | .10 | .104 | 1.54 | .23 | .058 | 0.17 | .69 | .007 |
| Note: *df* = 1, 25. | | | | | | | | | | | | |
